# Low specificity of HIV-testing on sputum specimens kept at ambient temperatures for 4 to 7 days: a blinded comparison

**DOI:** 10.1186/1472-6890-7-8

**Published:** 2007-09-19

**Authors:** Saidi M Egwaga, Timothy M Chonde, Mecky I Matee, Sayoki G Mfinanga, Prosper E Ngowi, Fred Lwilla, Frank GJ Cobelens

**Affiliations:** 1 National Tuberculosis and Leprosy Program, Dar-es-Salaam, Tanzania; 2 Central Tuberculosis Reference Laboratory, Dar-es-Salaam, Tanzania; 3 Muhimbili University College of Health Sciences, Dar-es-Salaam, Tanzania; 4 National Institute for Medical Research, Muhimbili Medical Research Centre, Dar-es-Salaam, Tanzania; 5 KNCV Tuberculosis Foundation, The Hague, The Netherlands; 6 Center for Infection and Immunity Amsterdam, Academic Medical Center, Amsterdam, The Netherlands

## Abstract

**Background:**

HIV testing on sputum using the QraQuick HIV1/2^® ^assay has high sensitivity and specificity, and holds promise for application in tuberculosis surveys. Its performance under conditions that may occur during surveys in resource-poor countries is however, unknown. We assessed, in a blinded comparison with HIV serum testing, the sensitivity and specificity of the OraQuick^® ^assay for detecting HIV antibody in sputum specimens kept at ambient temperature for up to 7 days, with and without decontaminant.

**Methods:**

Paired sputum and blood specimens from consecutively diagnosed smear-positive tuberculosis patients were tested with OraQuick^® ^and 2 HIV-1/2 ELISA's. Sputum was tested within 24 hours of collection, split into 2 aliquots with and without addition of cetylpyridium chloride, and tested again after 4 and 7 days.

**Results:**

Complete data was available for 377/435 (87%) enrolled patients; 132 (35%) tested HIV positive on serum. The sensitivity of the sputum test was 94.7% (95% CI 89.4–97.8) on day 1, 93.2% on day 4 and 92.9% on day 7. The specificity was 92.9% (95% CI 88.9–95.8) on day 1, and declined to 76.7% on day 4 (p < 0.001) and to 62.7% on day 7 (p < 0.001). Adding cetylpyridium chloride further decreased the specificity to 67.8% on day 4 (p = 0.04) and to 49.6% on day 7 (p = 0.004).

**Conclusion:**

Transportation of sputum specimens at ambient temperatures for 4 days or more, and addition of decontaminant, strongly affect the specificity of the OraQuick^® ^assay. Unless applied within one day, this assay is not suitable for estimation of HIV-prevalence among tuberculosis patients in survey settings.

## Background

The HIV epidemic has strongly increased tuberculosis incidences, in particular in sub-Saharan Africa [1]. For planning and evaluation of control efforts it is important to monitor the proportion of tuberculosis patients that is HIV-infected [2,3]. In settings with generalized HIV epidemics, as in most of Sub-Saharan Africa, the preferred method is surveillance based on routine HIV testing of every tuberculosis patient [3]. As long as access to HIV testing and antiretroviral treatment is limited [4], many countries need to rely on repeated surveys in which randomly selected tuberculosis patients are tested for HIV infection [3,5,6]. In such surveys, serum HIV testing poses problems of feasibility and requires informed consent, with the risk of non-participation and associated selection bias [3]. An attractive alternative is to detect HIV antibodies in sputum specimens [7,8]. This can be added onto surveys of tuberculosis patients in which sputum is collected, in particular for drug susceptibility testing. HIV testing can be centralized in one or few laboratories, and since sputum is already collected for other purposes, informed consent would not be needed, provided that anonymity and unlinking are safeguarded [8,9]. In order to be useful for this purpose, HIV testing on sputum should have sufficient sensitivity and specificity under conditions that apply in tuberculosis drug resistance surveys in high-prevalence countries. These include transit times between collection and processing of sputum specimens of up to 4 and sometimes to 7 days, specimen transport at high ambient temperatures and, if prolonged transit is expected, addition of cetylpyridiniumchloride (CPC) or cetylpyridiniumbromide (CPB) to minimize bacterial contamination [10,11].

In an evaluation on clinical specimens, the OraQuick HIV-1/2^® ^Assay, a lateral flow test that has been FDA approved for HIV testing on oral fluids, detected HIV antibodies in sputum with 98.4% sensitivity and 98.3% specificity on the day of collection [7]. Although specificity remained unaffected, sensitivity declined to 94.0% when specimens were tested more than 72 hours after collection but stored refrigerated. Based on these results the OraQuick test was used in a drug resistance survey among tuberculosis patients in Botswana [8].

We assessed the impact of prolonged storage at ambient temperatures and of addition of CPC on the diagnostic accuracy of the OraQuick test for detection of HIV antibodies in sputum samples.

## Methods

### Study design

In a prospective study among smear-positive tuberculosis patients in Dar-es-Salaam, Tanzania, the sensitivity and specificity of the OraQuick HIV antibody test on sputum specimens was assessed on the day of collection and 4 and 7 days later, with and without CPC, in blinded comparison using the result of two HIV enzyme immunoassays on serum as the reference standard.

### Participants

The study was conducted in six tuberculosis facilities in Dar-es-Salaam that diagnose and treat tuberculosis mainly on an outpatient basis. Eligible for inclusion were all patients aged ≥ 15 years who were consecutively diagnosed with sputum smear-positive tuberculosis during the period 1 November – 31 December 2004. There were no exclusion criteria. The study period was chosen to include approximately 400 patients. This sample size was based on the assumptions of 33% HIV prevalence among included patients and an observed sensitivity 94%, and on the requirement to estimate this sensitivity with a lower boundary of the 95% confidence interval (CI) of 90%. With this sample size, the lower boundary of the 95% CI for an observed specificity of 98% would be 94%.

### Specimen collection

Of each included patient, one sputum specimen and one blood specimen were collected. Sputum specimens were collected in sterile universal glass containers following standard procedures and transported to the laboratory on the same day [10]. Approximately 5 ml of venapuncture blood was transported to the laboratory within four hours of collection.

### Specimen processing and testing

Detection of HIV antibodies in sputum was done at the Central Tuberculosis Reference Laboratory (CTRL), and HIV serum testing in the Department of Microbiology and Immunology of the Muhimbili University College of Health Sciences (MUCHS), both in Dar-es-Salaam. Sputum samples were tested for HIV antibodies using the OraQuick HIV-1/2^® ^Assay on the day of collection (day 0) and then apportioned into two aliquots. One aliquot was placed into a sterile universal glass bottle containing an equal volume of CPC 1.0% while the other was kept in a non-CPC containing bottle [10]. Both aliquots were stored at ambient temperature until 96 to 120 hours after collection (day 4) when the specimens were tested using the OraQuick assay. These samples were then stored at ambient temperature until 168 to 192 hours after collection (day 7) and tested again. OraQuick testing was performed by two designated laboratory technicians following the manufacturer's recommendations (OraSure Technologies Products, Bethlehem, PA 18015, Ph. 610.882.1820). Briefly, the porous flat pad of the OraQuick device was stirred in the sputum specimen for at least 60 seconds, placed in the developer vial and read after 20 to 60 minutes. If the control line was not visible, a test was considered uninterpretable. If no lines appeared and the sputum was viscous, an equal volume of normal saline was added to dilute the sputum and the test was repeated using a fresh device [7].

Detection of IgG HIV antibody in serum was done using Vironostika HIV Uni-Form II Ag/Ab (BioMerieux, Boxtel, The Netherlands) and reactive samples were retested by Vironostika HIV Uni-Form II Plus O (BioMerieux, Boxtel, The Netherlands). Samples reactive on both tests were considered to contain IgG anti HIV antibodies. The technicians performing the sputum assay were blinded to the results of the serum assays, and vice versa.

Two weeks after the start of the study, data monitoring by one of the authors (FGC) revealed a remarkable discrepancy in sputum testing results between day 0 and day 4 that suggested declining specificity over time. We hypothesized that this decline in specificity was an artifact of our study design, i.e. was due to cross-contamination between sputum specimens resulting from difficulties in splitting the viscous material. To be able to assess possible cross-contamination during the splitting procedure on day 0, all sputum specimens received from 29 November through 12 December were left un-split and tested without CPC only. The technicians performing the sputum assay were not informed about the reason for this temporary change in procedure.

### Ethical issues

The study was conducted in compliance with the Helsinki Declaration [12]. Ethical clearance was granted by the Tanzania National Institute for Medical Research. Written informed consent was obtained from each patient prior to enrolment after explaining the objectives of the study, risks and benefits of participation. Study patients could choose to either or not obtain the result of their serum HIV test. Pre-test and, in case the participant opted to obtain the result, post-test counseling for HIV testing was done according to national guidelines. If informed of their test result, HIV positive patients were referred to the nearest HIV clinic for further management.

### Data management and analysis

Data was entered in duplicate in MS-Excel (Microsoft Corp., Seattle WA) and discrepancies were checked against the forms and laboratory records. Analysis was done using Stata v8 (Stata Corp., College Station TX). Sensitivity was calculated as (number positive on sputum/number positive on serum)*100. Specificity was calculated as (number negative on sputum/number negative on serum)*100. Testing for significance of categorical variables was with the Chi-squared test or the 2-sided Fisher's exact test, and of continuous variables with the t-test or Wilcoxon's rank sum test as appropriate. P-values < 0.05 were considered significant.

## Results

**Figure 1:**
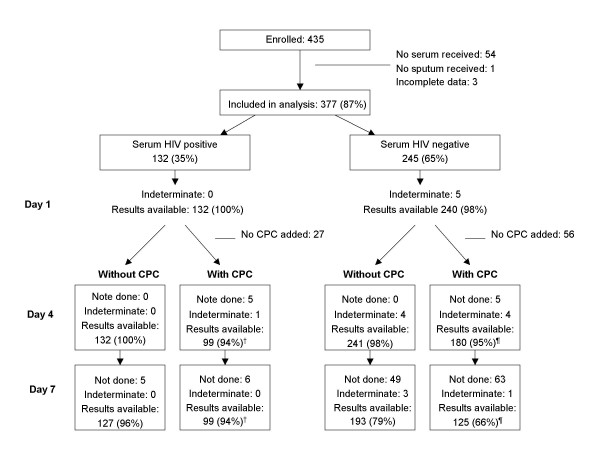
**Numbers of patients enrolled and numbers of sputum specimens available for the analyses**. Numbers of patients enrolled and numbers of sputum specimens available for the analyses by HIV ELISA results on serum (standard test), day of testing and addition of cetylpyridiniumchloride (CPC). Data for day 1, day 4 and day 7 refer to HIV testing on sputum specimens. Percentages refer to proportion of specimens with available test result out of number of patients tested positive or negative with the HIV ELISA on serum. †: Percentage of 105 specimens with CPC added. ¶: Percentage of 189 specimens with CPC added.

A total of 381 blood samples and 434 sputum samples were obtained from 435 patients. Complete data were available for 377 (87%, figure 1). Of these, 255 (68%) were between 15 and 34 years and 253 (67%) were male. There were no differences in age and sex between included and not included patients, but the proportion included varied significantly by clinic (Table 1). HIV results on sputum specimens without CPC were available for all 377 patients on day 1 and day 4. Due to insufficient material left for testing, 54 (14%) specimens were not tested on day 7 (figure 1). Eighty-three specimens had not been split on day 1 and were evaluated without CPC, and there were additional losses of sputum specimens with CPC for day 7. Therefore, results were available on days 4 and 7 with CPC for 284/377 (75%) and 225/323 patients (70%), respectively. Because some sputum specimens that tested negative on day 4 had been erroneously discarded. the proportion of sputum specimens that were available for testing on day 7 was lower if the day 4 test was negative than if it was positive, both without CPC (63% versus 93%, p < 0.01) and with CPC (50% versus 95%, p < 0.01). The proportion of indeterminate sputum results was less than 1.5% on any testing occasion.

**Table 1:** Characteristics of 377 included and 58 excluded patients with smear-positive tuberculosis

	Included N = 377	Not included N = 58	P
Age groups (years)			0.18
15–24	96 (25%)	15 (26%)	
25–34	159 (42%)	20 (35%)	
35–44	55 (14%)	16 (27%)	
45–54	42 (11%)	3 (5%)	
55–64	13 (4%)	1 (2%)	
65+	12 (3%)	3 (5%)	
Age (mean, Sd)	32.5 (12%)	33.7 (12%)	0.40
Male	253 (67%)	39 (67%)	0.98
Female	124 (33%)	19 (33%)	
Clinic of inclusion			< 0.01
Tambukareli	32 (9%)	1 (2%)	
Temeke	41 (11%)	9 (16%)	
Amana	102 (27%)	11 (20%)	
Mnazi Mmoja	45 (12%)	23 (40%)	
Tandale	58 (15%)	12 (21%)	
Mwananyamala	99 (26%)	1 (2%)	

Of the 377 patients in the analysis, 132 (35%) had a positive HIV serum result. The sensitivity of the sputum HIV test on day 1 was 94.7% (95% CI 89.4–97.8), and showed no significant change over time, neither with nor without adding CPC (figure 1). The specificity of the sputum HIV test on day 1 was 92.9% (95% CI 88.9–95.8). It decreased significantly over time, both when compared to day 1 (day 4 and 7, both with and without CPC: p < 0.01 for each comparison) and for day 7 versus day 4 (without and with CPC, p < 0.01 for both; Table 2). The specificity with CPC was significantly lower than without CPC, both on day 4 (67.8 vs 76.7%, p = 0.04) and on day 7 (49.6 vs 62.7%, p < 0.01).

**Table 2:** Sensitivity and specificity of HIV testing on sputum at various days, with and without CPC

	**HIV serum ELISA (Standard)**			
**HIV on sputum**	**Positive**	**Negative**	**Sensitivity (95% CI)**	**Specificity (95% CI)**

**Day 1**			94.7% (89.4–97.8%)	92.9% (88.9–95.8%)
Positive	125	7		
Negative	7	233		
Total	132	240		
**Without CPC**				
**Day 4**			93.2% (87.5–96.8%)	76.7% (70.9–81.9%)
Positive	123	56		
Negative	9	185		
Total	132	241		
**Day 7**			92.9% (87.0–96.7%)	62.7% (55.5–69.5%)
Positive	118	72		
Negative	9	121		
Total	127	193		
**With CPC**				
**Day 4**			93.9% (87.3–97.7%)	67.8% (60.4–74.5%)
Positive	93	58		
Negative	6	122		
Total	99	180		
**Day 7**			94.9% (87.5–97.9%)	49.6% (40.5–58.7%)
Positive	94	63		
Negative	5	62		
Total	99	125		

We tested the hypothesis that the observed decline in specificity was due to cross-contamination between sputum specimens during the splitting procedure by comparing sensitivity and specificity of the sputum test over time in the 83 specimens that were not split on day 1, and were tested without CPC on days 4 and 7. These specimens showed no differences in sensitivity when compared with split specimens, neither on day 4 (p = 0.89) nor on day 7 (p = 0.74; Table 3). On day 4 the specificity was lower on split than on unsplit samples (73.7 vs 87.3%, p = 0.03). On day 7 the specificity was also lower on split samples (59.3 vs 72.9%), but the difference remained non-significant (p = 0.09).

**Table 3:** Sensitivity and specificity of HIV testing on sputum without CPC, comparing split and unsplit specimens

	**HIV serum ELISA (Standard)**			
**HIV on sputum**	**Positive**	**Negative**	**Sensitivity (95% CI)**	**Specificity (95% CI)**

**Split specimens**				
**Day 4**			93.3% (86.7–97.3%)	73.7% (66.7–79.8%)
Positive	98	51		
Negative	7	135		
Total	105	186		
**Day 7**			93.3% (86.6–97.3%)	59.3% (50.8–67.4%)
Positive	97	59		
Negative	7	86		
Total	104	145		
**Unsplit specimens**				
**Day 4**			92.6% (75.7–99.1%)	87.3% (75.5–94.7%)
Positive	25	7		
Negative	2	48		
Total	27	55		
**Day 7**			91.3% (72.0–98.9%)	72.9% (58.2–84.7%)
Positive	21	13		
Negative	2	35		
Total	23	48		

Of 377 included sputum specimens, 321 (85.1%) had been processed and tested by one technician, 32 (8.5%) by another. For 24 specimens the technician had not been recorded. Mean sensitivity or mean specificity of the sputum HIV test showed no significant differences between the two technicians (data not shown).

## Discussion

Our results show that HIV testing using the OraQuick HIV-1/2^® ^Assay on sputum specimens of smear-positive tuberculosis patients had more than 90% sensitivity and specificity for detecting HIV-antibodies if done on the day of sputum collection. Although slightly lower than reported from a study using clinical specimens of tuberculosis patients in Botswana [7], these may be considered high enough for this test to be applied in surveys of HIV-infection among tuberculosis patients in settings with high expected HIV-prevalence. For example, with the prevalence of HIV infection (35%) and specificity on day 1 (93%) observed in the current study, the positive predictive value of the sputum HIV test can be calculated as 88%, i.e. no more than 12% of positive sputum tests would be false-positive. This would still provide a reasonably valid estimate of the HIV prevalence in this patient population.

When the sputum test was done 4 days after collection, the specificity decreased significantly to 77% for specimens without and to 68% for specimens with CPC. In a setting of 35% HIV prevalence, this would correspond to proportions false-positive of 31 and 39%, respectively. With 10% HIV prevalence, the minimum prevalence at which WHO allows the use of sputum HIV testing [3], the corresponding proportions false-positive would be as high as 69% and 75%, whereas they would still be 19% and 25% at a very high prevalence of 50%. We think that such false-positive rates are too high for surveillance purposes. Delay to 7 days decreased the specificity even further to only 63% and 50%, respectively, with further increases in corresponding proportions false-positive.

Our results could also mean that the sensitivity of the sputum test was higher than that of the reference standard. It is however, unlikely that the high sensitivity of the combined serum enzyme immunoassays used in this study (99.7% in the same setting, 95% CI 98.2 – 100%) is surpassed by that of the sputum test [13].

From the presented data it seemed that adding CPC further decreased the specificity. However, this reflects at least in part the higher specificity on specimens that were not split during a two-week period when all unsplit specimens had been tested without CPC. The difference in specificity between split and unsplit specimens may also point to a possible explanation for the decrease in specificity over time. During the splitting procedure cross-contamination of specimens may have occurred whereby HIV antibody results became false-positive upon subsequent testing. However, the differences in specificity between split and unsplit specimens were only marginally significant. In addition, there was a further and significant decrease in specificity between day 4 and day 7, which is unlikely to be due to cross-contamination during the splitting procedure.

Another source of bias in the specificity estimate could be that the 15–20% specimens that were not tested on day 7 were more often negative on day 4 than were the specimens that were tested on day 7. If all specimens that were not tested on day 7 would have tested HIV negative, the specificity on day 7 would be 70.2% (170 of 242) for specimens without and 66.5% (125 of 188) for specimens with CPC. Thus, the difference between day 4 and day 7 may be partially explained by this bias.

However, these potential biases do not affect our conclusions of a remarkable decrease in specificity over time. The cause of this decrease is unclear. An earlier evaluation on clinical sputum specimens in Botswana observed no such decrease over a 3-day period, but specimens were kept refrigerated [7]. It may thus be that when kept at ambient temperatures, as expected in a countrywide anti-tuberculosis drug resistance survey, specimens change in such a way that cross-reacting substances are generated. In addition, liquefaction of the sputum specimen over time could contribute to false-positive readings, perhaps due to different absorption by the testing device. This poses a research question for further study.

## Conclusion

Unless testing within one day after sputum collection can be guaranteed, HIV testing on sputum using the OraQuick HIV-1/2^® ^Assay is not suitable for use in tuberculosis drug resistance surveys for the purpose of obtaining a valid estimate of the HIV-prevalence among tuberculosis patients.

## Competing interests

The author(s) declare that they have no competing interests.

## Authors' contributions

SME, FL and FGC designed the study. TMC and SGM organized data collection. TMC and EN did the HIV antibody assay on sputum. MM was responsible for HIV testing on serum. FGC analyzed the data. SME, MM and FGC wrote the draft paper. All other authors have been involved in critical revision of the paper. All authors have given final approval of the version to be published.

## Pre-publication history

The pre-publication history for this paper can be accessed here:


